# The urothelium: a multi-faceted barrier against a harsh environment

**DOI:** 10.1038/s41385-022-00565-0

**Published:** 2022-09-30

**Authors:** Nazila V. Jafari, Jennifer L. Rohn

**Affiliations:** Department of Renal Medicine, Division of Medicine, University College London, Royal Free Hospital Campus, London, UK

## Abstract

All mucosal surfaces must deal with the challenge of exposure to the outside world. The urothelium is a highly specialized layer of stratified epithelial cells lining the inner surface of the urinary bladder, a gruelling environment involving significant stretch forces, osmotic and hydrostatic pressures, toxic substances, and microbial invasion. The urinary bladder plays an important barrier role and allows the accommodation and expulsion of large volumes of urine without permitting urine components to diffuse across. The urothelium is made up of three cell types, basal, intermediate, and umbrella cells, whose specialized functions aid in the bladder’s mission. In this review, we summarize the recent insights into urothelial structure, function, development, regeneration, and in particular the role of umbrella cells in barrier formation and maintenance. We briefly review diseases which involve the bladder and discuss current human urothelial in vitro models as a complement to traditional animal studies.

## Introduction

The epithelial cells that line mucosal surfaces form a barrier between the internal and external environments with a continuous layer of tightly linked cells. The harsh conditions at the external-facing surfaces of organs and tissues require a structurally robust epithelium that maintains a barrier to the outer environment^1^. Mucosal epithelia at sites such as the gastrointestinal (GI) tract, the respiratory and the genitourinary tract must strike a balance between facilitating a selective transport while also forming a barrier with restricted paracellular transport^2^.

Most epithelia share common core functions including protection, sensation, transport, secretion, clearance, and repair, and they protect organs by providing a unique interface for each organ to inhabit. They also form diffusion barriers that separate distinct compartments, often from the external environment, with diverse permeability, which can be classified as either leaky or tight^3^. Epithelial cells sense their environment, and many facilitate active and passive transcellular and passive paracellular transport. Ions, water, and other substances transported by epithelia aid luminal surface hydration, while mucins assist in surface lubrication, supporting mucosal homeostasis. Fronting such a hostile environment, the epithelia must inevitably regenerate. While the location of the stem cell compartment varies among epithelia, it is often located at the base, allowing cell migration toward the lumen^3^.

The urothelium (sometimes referred to as *uroepithelium*) is a stratified, transitional epithelium that lines the renal pelvis, ureters, bladder, and proximal urethra^4^. This mucosal surface layer plays an important barrier role, preventing absorption of urine’s toxic substances such as acid and urea and defending against pathogen entry from the external environment^5–7^. The urothelium consists of three cell types: basal, intermediate, and superficial cells, also known as umbrella cells or facet cells^8^. The basal cells are the most undifferentiated urothelial cell type, located at the basement membrane of the lumen and serving a progenitor role. The intermediate cells are highly proliferative, forming multiple cell layers depending on the species. In times of injury or infection, intermediate cells are responsible for rapidly regenerating the urothelium. On the apical surface, fully differentiated umbrella cells are responsible for maintaining the impermeability and high-resistance barrier function of the urothelium^4,8,9^. In this review we will discuss the structure and function of the urothelium and recent advances in developing in vitro models to study host-pathogen interaction. We focus on human systems and, if not otherwise indicated, statements refer to the human context.

## The urothelium: structure and function

Here, we discuss in detail the three main cell types that comprise the bladder urothelium.

### Basal cells

**Fig. 1:**
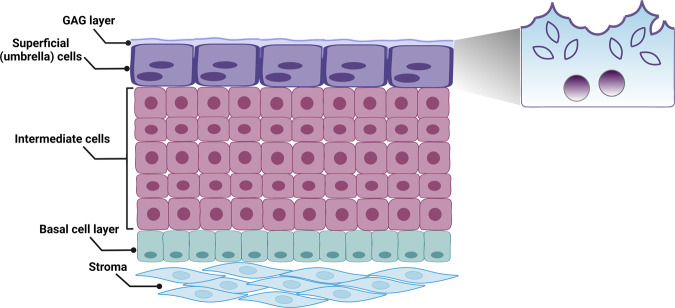
Bladder urothelium cell layers. The urothelium is composed of three cell types: basal cells, intermediate cells, and superficial or umbrella cells. Umbrella cells are covered by an apical membrane plaque comprised of uroplakin proteins at the luminal surface, and contain a large pool of subapical vesicles.

The basal cell layer is positioned along the basement membrane (Fig. 1); they are the smallest of the urothelial cells (5–10 µm in diameter) but constitute the most abundant cell population in adult urothelium^10^. They are attached directly to the basement membrane via hemidesmosomes^11–13^ and to the overlying intermediate cells by desmosomes. As discussed in more detail in a later section, it has been proposed that basal cells potentially harbor a subset of urothelial stem cells providing lifelong regeneration of the urothelium^9^. In a study using single-cell transcriptomic analysis of mouse bladder urothelium, a cluster of cells was distinguished expressing the marker gene Abnormal Spindle Microtubule Assembly (ASPM); genome-wide analysis suggested this ASPM^+^ expression could implicate these basal cells as stem/progenitor cells^14^. However, studies also suggest that both basal and intermediate cells are undifferentiated precursor cells with the ability to undergo a programmed differentiation into umbrella cells during development and in the adult urothelium^6,15^.

**Fig. 2:**
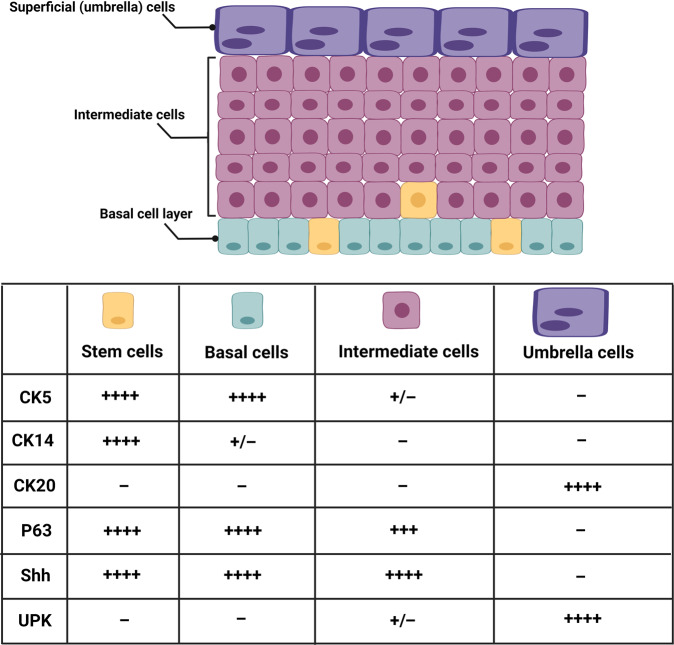
Development and regeneration of the urothelium. The cell types and differentiation markers expressed during the urothelium development and in response to injury.

Basal cells are distinguished by expression of high levels of cytokeratin-5 (CK5), p63^16^, and the signalling molecule Sonic hedgehog (Shh)^17^. Together with intermediate cells, they express CK17^6^ but are negative for uroplakins (UPK) and CK20^18^ (Fig. 2).

### Intermediate cells

The cell layer directly above the basal cells is referred to as the intermediate cell layer and depending on the species, this layer can range from one to several layers thick^5^ (Fig. 1) (e.g., there are approximately five intermediate cell layers in humans and one in rodents)^19,20^. The intermediate cells are larger in diameter than basal cells (∼20 μm) and are attached to the adjacent cell layers and one another via desmosomes^5,20^. They differ from the basal cells based on their expression of UPK and lack of CK5^19,21^, similar to umbrella cells (UPK^+^, CK5^−^), but unlike umbrella cells they additionally express p63 (UPK^+^, p63^+^, CK5^−^)^10^ (Fig. 2). Basal and intermediate cells also express CK17 which is completely absent in umbrella cells^6^. Although intermediate cells express tight junction-associated proteins such as claudins^22^, and the E-cadherin epithelial cell adhesion protein, they do not seem to form morphologically discernible tight or adherens junctions^23^.

### Umbrella cells

The superficial umbrella cells form a single layer of terminally differentiated and highly specialized cells that directly face the luminal surface^15^ (Fig. 1). These cells are large, hexagonal in shape, highly polarized, and in some species, multinucleated (e.g., rat and guinea pig)^24^. Umbrella cells are long-lived (∼200 days in rodents)^25^ and can range in size from 25 to 250 μm depending on the bladder distension level^20,22^. In the relaxed state, superficial cells form a dome-shaped structure at the apical pole, and can also cover multiple underlying intermediate cells, hence the name umbrella cells^20,22^. In contrast, when the bladder is filled, they become large and flattened (see section: maintaining the barrier during mechanical changes). Umbrella cells are attached to sub-superficial cell layers via desmosomes, while tight junctions localized between superficial cells aid in forming the high-resistance barrier function^22,26^. The umbrella cell layer is the only urothelial layer that forms detectable tight and adherens junctions, which are principally responsible for barrier function by sealing the intercellular space between the adjacent cells^23^. Urothelial tight junctions are comprised of tight junction protein 1 (ZO-1), occludin, claudin-4, 8, and 12^26,27^.

Four major UPK are synthesized by umbrella cells in mammals, which include UPK1A, UPK1B, UPK2, and UPK3A. They comprise a small family of transmembrane proteins and form a hexagonal crystalline lattice at the apical membrane^19,28,29^. Together, the urothelial plaque and junctional complexes establish high electrical resistance and a highly effective permeability barrier, both of which regulate water and ion passage from urine to the underlying tissue^26,27,30,31^. Single-cell transcriptomic analysis of mouse urothelium revealed a novel cluster of cells enriched for Plxna4; as these cells also highly expressed UPK3, they appeared to be umbrella cells. However, they were negative for CK20, which is a urothelial differentiation marker. The authors therefore concluded that the Plxna4^+^ urothelial cell cluster is a special type of urothelial cells^14^.

Another distinguishing feature of umbrella cells is the presence of subapical discoidal/fusiform-shaped vesicles (DFVs) contributing to the plasticity in urothelial cell surface area through a regulated process of endocytosis/exocytosis^20,32,33^. A major functional role of the DFVs is to fuse with the apical membrane of the umbrella cells and release UPK and other proteins in response to bladder filling. This adjusts the permeability barrier and allows the expansion of the urothelium^34–36^. During emptying of the bladder, the reverse process occurs, causing the decrease of urothelial surface area. Umbrella cells also express high concentrations of CK20, this protein contributes to a cytokeratin network located below the apical surface of the superficial cells which guides DFVs to the surface^37^.

In addition to the literature discussed above, the Human Protein Atlas also contains useful and evolving information about the urinary bladder-specific proteome (Human Protein Atlas proteinatlas.org)^38^.

## Urothelial development

Although the term “urothelium” is used to describe the epithelial lining of both upper and lower urinary tracts, the ontogeny of the urothelium varies. The proximal urethral and bladder urothelium is derived from the endoderm, whereas the urothelia lining the ureters and renal pelvis are mesoderm-derived^19,39^. Irrespective of the origin, the primordial urothelium starts off as a single layer of immature, cuboidal epithelial cells. These cells undergo cell division under the direction of ligands produced by the stroma, and ultimately differentiate into three defined layers of basal, intermediate and umbrella cells as discussed above^16,40–42^.

### Regeneration and repair of the urothelium

Under homeostatic conditions, the adult urothelium is mitotically quiescent, and turnover is very slow^5,43,44^. However, in response to injury, there is a marked upregulation in urothelial proliferation resulting in rapid repair and regeneration, terminating with a completely restored, morphologically normal appearance within a few days to weeks^16,17,22,30,40,45–50^. Urothelial renewal depends on input from both the stroma and the urothelium; required signalling pathways include those regulated by ﻿bone morphogenetic protein 4 (BMP4), non-canonical and canonical Wnt, Delta-Notch, the epithelial cell-specific transcription factor ELF3, several growth factors, retinoids, Sonic hedgehog (Shh and GLI1), and TP63 (tumor promoter 63 kDa, p63, or Trp63)^10,16,17,40,50–54^.

Following acute urothelial injuries by chemical exposure (e.g., chitosan, cyclophosphamide, protamine sulfate, saccharin), surgical damage (for example during augmentation cystoplasty or focal mucosal resection) or infection with uropathogenic microbes, the urothelium starts repairing almost immediately. Uropathogenic *Escherichia coli* (UPEC), the primary cause of urinary tract infection^55^ (UTI), initiates a UTI using FimH located at the tip of the Type 1 pili^56^, mediating adhesion to *N-*linked carbohydrates covalently attached to UPK1A proteins expressed at the apical surface of umbrella cells^57^. FimH-mediated interactions with the urothelium stimulate umbrella cell exfoliation which in turn prompts proliferation of the remaining urothelial cells and, ultimately urothelial regeneration^50^.

The basal cell layer in other stratified epithelial cells serves as a stem cell population to maintain epidermal growth and renewal. Therefore, urothelial progenitors may also be located in the basal cell layer (Fig. 2). Basal cells express high levels of CK5, but studies have identified a subset of basal cells also expressing CK14. Under homeostatic conditions, these cells are the only mitotically active cells and have been identified as long-term label-retaining cells^58^. Several studies have investigated urothelial renewal during homeostasis and regeneration following insults of a chemical, surgical or bacterial nature. Papafotiou et al. identified a rare subset of basal cells in mice embryonic bladder that expresses *Krt14* (encoding CK14) and exhibits progenitor properties, based on genetic fate-mapping in vivo and greater self-renewal capacity in vitro^49^. This study revealed that a single round of cyclophosphamide-induced injury stimulates a local proliferation of CK14^+^ basal cells; however, after several consecutive treatments, CK14^+^ cells were found in all three urothelial layers. Another mouse study identified a transient progenitor population during embryogenesis but reported that the uroplakin-positive intermediate cells were the source of both intermediate and umbrella cells in juvenile and adult bladders^16^. Similarly, a study on the developing mouse ureter concluded that the umbrella and basal cells of the primordial urothelium are mainly derived from uroplakin-positive intermediate progenitor cells^59^.

A genetic fate-mapping study of intrarenal urothelial development in mice revealed that progenitor cells expressing *Krt5* (encoding CK5) can give rise to uroplakin-expressing cells^60^. However, they concluded that the differentiation of CK5^+^ cells into uroplakin-expressing cells was chiefly restricted to early time periods, as juvenile and adult CK5^+^ cells showed lineage restriction. Other mouse model studies have indicated that CK5^+^CK14^+^ basal cells expand in response to urothelial injuries and therefore are the progenitor cells of all urothelial lineages^58,61^. Schafer et al. showed that in a mouse surgical bladder injury model following augmentation cystoplasty, CK5-expressing basal cells repopulate all lineages of the urothelium. However, the repair was surgical procedure-dependent, as repair of focal mucosal defects instead employed CK5 basal cell repopulation in parallel with intermediate cells, which express UPII to regenerate themselves and also give rise to umbrella cells in neotissues^62^.

Several studies also observed that in response to UPEC infection, the CK5^+^ and Shh^+^ basal cells, and possibly intermediate cells sharing the same phenotype, proliferate and give rise to other cell types^17,40,48^. Whether the results of these rodent models of studies are relevant to human urothelial development/repair remain unknown, but they do indicate that the type and extent of injury likely defines the urothelial progenitor populations that are responsible for regeneration.

## Urothelial differentiation markers

Although there are many potential urothelial differentiation markers, only a relatively small number have been classified and of these, most are in mice. Such markers include UPK, cytokeratins, and signalling/transcription factors such as Shh, Tp63, and FOX2A (Forkhead box protein A2). In this review we discuss UPK and cytokeratins.

### Uroplakins

**Fig. 3:**
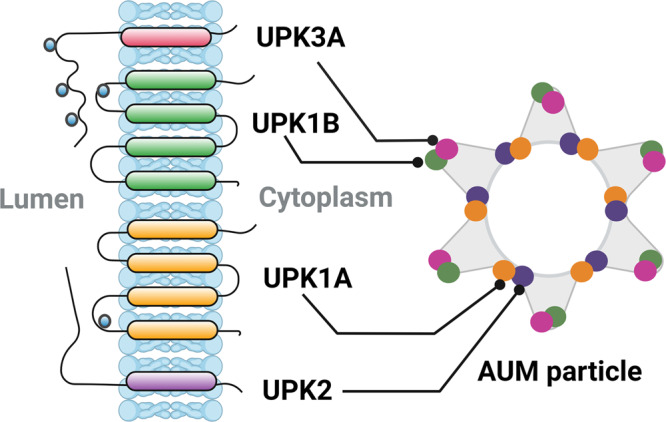
Uroplakins form asymmetric unit membrane (AUM) particles. Uroplakins embedded in a lipid bilayer are arranged in 16-nm AUM particles. The AUM inner ring is comprised of UPK1A-UPK2 heterodimers, and the outer ring is formed by UPK3A-UPK1B heterodimers. UPK3A and UPK2 moieties interact to form heterotetradimers.

As mentioned above, UPK are differentiation-specific tetraspanin membrane proteins mainly associated with the umbrella cells^63^. To date, five UPK have been identified including UPK1A, UPK1B, UPK2, UPK3A, and UPK3B. They are the major constituents of the urothelial plaques and asymmetric unit membrane (AUM), the characteristic apical membrane of the superficial layer^19,21^. Initially, UPK1A dimerizes with UPK2, and UPK1B with UPK3A and 3B forming heterodimers^63–66^. The importance of dimerization is underscored by the inability of the UPKs, when expressed individually, to exit the endoplasmic reticulum and reach the membrane^67^. The heterotetradimers are formed following the interaction of UPK2 and UPK3A moieties of the heterodimers. Following assembly within the endoplasmic reticulum and the Golgi, six heterotetradimers arranged in inner and outer rings^68^ form a 16 nm AUM particle or plaque (Fig. 3), packaged into DFVs and delivered to the apical membrane^69^. The AUM particles are also capable of changing their arrangement in response to mechanical changes such as bladder expansion and contraction so therefore undergo high renewal^70^. The urothelial plaque decorates up to 90% of the luminal surface and confers transcellular resistance, restricting permeability to water and solutes, and toxins^70,71^.

All UPKs have extensive exoplasmic domains resulting in thickening of the outer leaflet of the membrane, a feature that contributes to the permeability function of the urothelium^66^. The role of UPK in urothelial barrier function has been demonstrated by the fact that UPK3A knockout mice showed decreased urothelial barrier function, including increased water and urea permeability^72^.

Although UPK are considered to be markers for umbrella cells across species, in mice there is a population of intermediate cells that express UPK, in particular UPK3A^16,73^. These cells are usually located in the layer of cells immediately below the umbrella cells when the superficial layer is damaged. As another exception, UPK2 is expressed in both umbrella and intermediate cells of the mouse bladder^74^. In the human bladder, all UPKs are expressed specifically in the urothelium except UPK1B^19^. There is also an increasing appreciation that UPK are expressed in non-urothelial cells^75–78^.

### Cytokeratins

Urothelial distension and the increase of luminal surface area caused by bladder filling require a resilient mechanical support to resist extreme stretching forces. Cytokeratins are the best candidates among cytoskeletal filaments to protect urothelial cells against such mechanical stress^79,80^. Unlike other cytoskeletal filaments, the elasticity of the cytokeratins increases in response to tension. Once the strain is released, they are able to recover almost immediately and regain their original shape^79^.

The cytokeratins may be regarded as differentiation markers because cytokeratin isotypes are expressed by almost all epithelial cell lineages, and distinct cytokeratin expression profiles are associated with particular epithelial differentiation pathways. Furthermore, the expression of certain cytokeratin isotypes may be associated with a specific maturation stage. Therefore, these different aspects need to be considered when interpreting cytokeratin expression, which may be modulated according to the differentiation and/or pathological status of a tissue.

In humans, cytokeratins consist of more than 20 isotypes of proteins including type I (CK9-CK20) and type II (CK1-CK8). In all epithelial cells, intermediate filaments are composed of at least one type I and one type II cytokeratin, forming coiled-coil heterodimers which are expressed in a tissue- and differentiation-dependent manner^23,81^. The ratio of type I to type II is always 1:1, irrespective of the number of cytokeratins expressed in a particular epithelial cell^82,83^.

The urothelium is reported to express numerous cytokeratins including CK4, CK5, CK7, CK8, CK13, CK14, CK17, CK18, CK19, and CK20^37,81,84–88^. Cytokeratin expression in the urothelium varies and depends on its location. In mice, CK10 is expressed only in the urethral urothelium^86^, while CK6 in humans is exclusively expressed in the renal pelvis urothelium^84^. Moreover, cytokeratin expression or distribution differs between species^16,84^. In mice, CK5, CK14, CK20, and to lesser extent CK17 are the markers most often used to describe urothelial differentiation^16,17,89,90^. CK20 is only expressed in mouse umbrella cells^89^ and CK7 is reported to be solely expressed by a population of intermediate cells^91^, although there are studies showing that CK7 is expressed throughout the mouse urothelium^92^. While CK14 is only expressed in a small population of basal cells^49^, CK5 expression is detected in all cells located in the basal layer including those that are CK14 positive^16,17,39,49^. In addition, CK5 is expressed by most intermediate cells apart from cells that express UPK3A^16,39^.

In normal adult human urothelium, CK7, CK8, CK18, and CK19 expression has been observed throughout all urothelial cell layers. While CK17 and CK5 are basally expressed, CK20 is associated with umbrella cells^83,93,94^. Although CK13 is present in basal and intermediate cell layers, it is used as marker of the switch from basal cells to differentiated urothelial transitional cells^95^. Studies have reported that normal human cells in culture exhibit late/terminal cytodifferentiation when activated with PPARγ agonists, promoting a switch from a non-differentiated phenotype (CK14^+^, CK13^−^, CK20^−^) to a terminally differentiated transitional phenotype (CK14^−^, CK13^+^, CK20^+^)^96,97^.

## The urothelial barrier

The bladder urothelium is exposed to great osmotic and chemical gradients and mechanical changes as urine is produced, transported, stored, and voided from the bladder. Therefore, the urothelial barrier function is essential to maintain a high-resistance barrier for prolonged periods to the outside environment which includes excess water, ions, solutes, and metabolic waste products, preventing the diffusion of harmful urinary products into the underlying tissues and moreover, defending against pathogens. This barrier is complex and includes three components: the apical, the lateral, and the basal barrier. The apical membrane barrier is composed of UPK, in which AUM particles are assembled into hexagonal plaques forming a flexible apical barrier. The umbrella cell tight junctions form the lateral barrier, and the basal barrier consists of group of proteins including cadherin, claudins, and laminins^98^.

### Urothelial glycocalyx

The glycocalyx is a dense, gel-like meshwork that forms a physical barrier at the apical membrane of the umbrella cell layer^22,99^. Although prominent in enterocytes lining the gut and in endothelial cells, only a thin layer has been visualized in the urothelium using transmission electron microscope (TEM)^5,34,100,101^. The glycocalyx comprises membrane-bound glycoproteins and glycolipids, along with soluble components including galectins and proteoglycans. The glycosaminoglycans (GAG), composed of unbranched carbohydrates with repeating disaccharide units, are attached to a core protein to form a proteoglycan. The GAG layer consists mainly of heparin sulfate, dermatan sulfate, chondroitin sulfate, hyaluronic acid, and keratan sulfate. Chondroitin sulfate and hyaluronic acid, the two main components, play a central role in forming the barrier and in antibacterial defence^98^. There is convincing evidence that the glycocalyx may have intrinsic, nonspecific, and anti-adherence properties that protect against pathogens^102,103^.

### The apical junctional complex and regulation of paracellular permeability

**Fig. 4:**
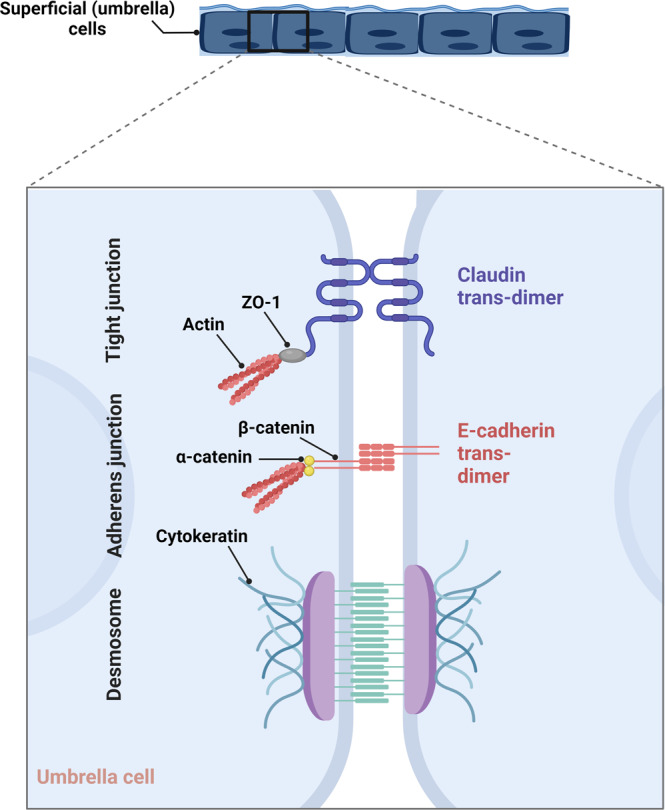
Apical junctional complex in umbrella cells. The junctional complex is comprised of the ringlike tight junction (TJ), adherens junction (AJ), and desmosomes. TJs are the most apical intercellular junctions. The key molecular components of TJs are claudins and occludin. TJ proteins in conjunction with AJ proteins (cadherins and catenins) form the urothelial junctional complex.

Apical junctions are specialized epithelial structures; as a hallmark of polarized epithelial cells, they play a crucial role in regulating paracellular transport^1,104,105^. Apical junctional complex (Fig. 4) is comprised of three components: (i) the apical tight junction or zonula occludens, which is formed by transmembrane claudins together with other membrane and cytoplasmic proteins, linked to the actin cytoskeleton; (ii) the zonula adherens (subjacent adherens junction) which is comprised of cadherins and associated catenins tethering the adherens junctions to the actin cytoskeleton; and (iii) the desmosomes or macula adherens, comprised of cadherin-like molecules (desmogleins and democollins) and their associated cytoplasmic proteins that mediate the desmosomes attachment to the intermediate filament cytoskeleton^1,105–107^.

Epithelial cell membranes form a barrier to macromolecules and hydrophilic solutes including ions and water; however, these molecules can potentially navigate the paracellular pathway at the cell junctions. The adherens junctions (AJ) and desmosomes are crucial to link intercellular adhesion to the actin or intermediate filaments cytoskeletons and connect adjacent epithelial cells together. Nevertheless, these junctions do not seal the paracellular pathway; this pathway is instead controlled by tight junctions (TJ), the component of the apical junctional complex bordering the lumen^3^.

The TJ is located at the apex of two adjacent cells, forming a continuous ring. TJs have two basic functions; first, they act as a “fence” separating the apical and basolateral membrane domains; in doing so, they restrict the lateral diffusion of membrane proteins and lipids^108–111^. Second, they possess a “gate” function which is responsible for regulating the paracellular diffusion of ions and other molecules between cells^112–116^. The TJ is composed of two families of transmembrane proteins, the claudins and occludins^117^, which form homotypic claudin-claudin and occludin-occludin complexes between cells. The complementary assembly of TJ strands between adhering cells creates a complex network of gaps and pores through which different ions and solutes are thought to diffuse^108,118–121^. Diffusion in the paracellular pathway varies with claudin types and is gated via different amino acids in the extracellular loops of claudins. The combination of claudin isoforms specifies the permeability of ions of different size and charge, which classifies the claudins into either “pore forming” or “barrier forming”^121,122^.

In the urothelium, umbrella cells are characterized by apical expression of transmembrane uroplakins that contribute to transcellular barrier function^65,71^, while the paracellular barrier is maintained by intercellular tight junctions with claudin proteins defining the paracellular permeability^117,123^. The permeability across the TJ differs in a cell type-specific manner. In the proximal tubule of the kidney, for example, the transepithelial electrical resistance (TEER) is ~200 Ω cm^2^, while the urothelial TEER is ~75,000 Ω cm^2^ in the presence of amiloride, which blocks transcellular sodium transport^110^.

Claudin 1 is ubiquitously expressed in most tissues^124^ and mainly acts as a barrier builder^125,126^. It is found in human urothelium^123,127,128^ along the basal and intermediate cell membrane but it is particularly enriched in the basal surface of the basal cell layer^129–131^. Claudin 2 forms a high-conductance cation-selective pore^120,132–134^ and is detected in the proximal tubule of the kidney and in the intestinal crypts, both of which are considered “leaky” epithelia^135–137^. Interestingly, in mouse bladder, claudin 2 is expressed in all three cell layers^26^. In the human urothelium, the expression of claudin 3 is necessary for the development of the umbrella cell terminal tight junction^27^. It is a ubiquitously expressed barrier-forming claudin^138^ which is restricted to the apicolateral plasma membrane of the umbrella cells in human^130,131,139^ and mouse urothelium^140^. Several studies indicate that claudin 4 is barrier-forming^141–147^ and is present in human and rodent urothelium, with its expression higher in umbrella and intermediate layers compared with the basal cells^26,123,128–131,139,148^. In humans, claudin 7 is distributed similarly to claudin 4^129–131,139^ with the exception of umbrella cells^123,129,148^. Claudin 8 is another barrier-forming claudin^149^ shown to localize primarily in the TJs of the umbrella cells in human and rodent bladders^26,150,151^. Zonula occludens-1 (ZO-1), also known as tight junction protein-1 and integral protein occludin, is also present at the TJ of umbrella cells^26,150,152^. Of note, there are reports indicating regional differences in the distribution of TJ-associated proteins. For instance, *CLDN1* and *CLDN4* mRNA levels are significantly higher in the human bladder trigone than in the dome^128^.

### Water and urea transport across the urothelium

The apical membrane of umbrella cells along with the TJ form a relatively impermeable barrier to the unrestricted diffusion of solutes and water^23^, but there is ion flux across the epithelium^153,154^. Sodium is the primary transported ion^110^, by a mechanism modulated by several molecular and physical factors^111,155–157^. Studies have shown that rabbit urinary bladder exhibits an extremely low permeability to ions with a TEER above 20,000 Ω cm^2^ in the quiescent state and ~75,000 Ω cm^2^ when transcellular sodium transport is blocked^110,150^. In addition, lipid bilayers have inherently low permeability to ions, so their diffusion across the membrane depends on the presence of ion channels^158^. It has been proposed that the mechanosensitive ion channels located in the apical membrane of umbrella cells which support transepithelial sodium ion flux may have a sensory role in normal micturition^159^.

The urothelium also expresses several aquaporins (AQPs), a family of 13 members that transport water or small solutes such as NH_3_, CO_2_, glycerol, and urea across cell membranes^160,161^. The AQPs can be divided into two subfamilies based on their function: the “orthodox” water transporting AQPs (AQP0, AQP1, AQP2, AQP4, AQP5, AQP6, and AQP8); and the aquaglycoporins (AQP3, AQP7, AQP9, and AQP10), which mediate the transport of water plus small uncharged solutes, such as glycerol, urea, and pyrimidines^162^. Several AQP family members are expressed in human urothelium^163^. The expression of AQP2 and AQP3 in rat ureter and bladder has been reported previously with AQP1 localized to endothelial cells^164^. Both AQP2 and AQP3 are located primarily at the basolateral membrane of umbrella cells and the plasma membranes of the intermediate and basal cells^164^.

In humans, transcripts for AQP3, AQP4, AQP7, AQP9, and AQP11 were detected in freshly isolated urothelia and normal human urothelial (NHU) cells in culture^163^. Strong AQP3 expression was apparent at the cell borders in basal and intermediate cells in both urothelium in situ and in vitro differentiated cells^163^. While expression of AQP3, AQP4, and AQP11 transcripts were consistent in bladder tissue and cultured urothelia, AQP9 was expressed in bladder tissue and differentiated NHU cultures, but not proliferative cultures. It has been suggested that the expression of AQP9 might be associated with terminal differentiation in transitional epithelia^165^.

Studies also have indicated that the bladder epithelium may play a modulatory role in water and salt homeostasis. In dehydrated rats, a significant upregulation of AQP2 and AQP3 proteins was observed, providing plausible evidence that AQPs are involved in water and solute transport^164^. Furthermore, urea transporters are expressed in the urothelium of bladder and ureters^166–170^. The urothelium also has pathways for ion reabsorption and aquaporin-independent water transport, although these pathways are unclear. Several studies have suggested that the urothelium can modify the composition of urine^171^, depending on the hydration conditions^167,172^ and/or bladder distension^173,174^.

### Maintaining the barrier during mechanical changes

The bladder urothelium must withstand an astonishing array of punishment as it is exposed to tremendous mechanical stretch forces, osmotic pressure and hydrostatic pressure, all while needing to maintain one of the least permeable barriers in the body. Investigations into the effects of bladder filling and voiding on the structure and function of the umbrella cell TJ have shown that filling promotes a significant increase in the perimeter of the TJ ring, which is rapidly reversed back upon voiding^150^. When rabbit urothelium mounted in an Ussing chamber is stretched, there is a significant drop in overall TEER and TJ-associated resistance, leading to umbrella cell TJs being leakier to ions. Remarkably, the integrity of the urothelial barrier is maintained even with a ten-fold drop in TEER, as no significant leakage of biotin, fluorescein, or ruthenium is detected across the urothelium under these conditions^150^.

**Fig. 5:**
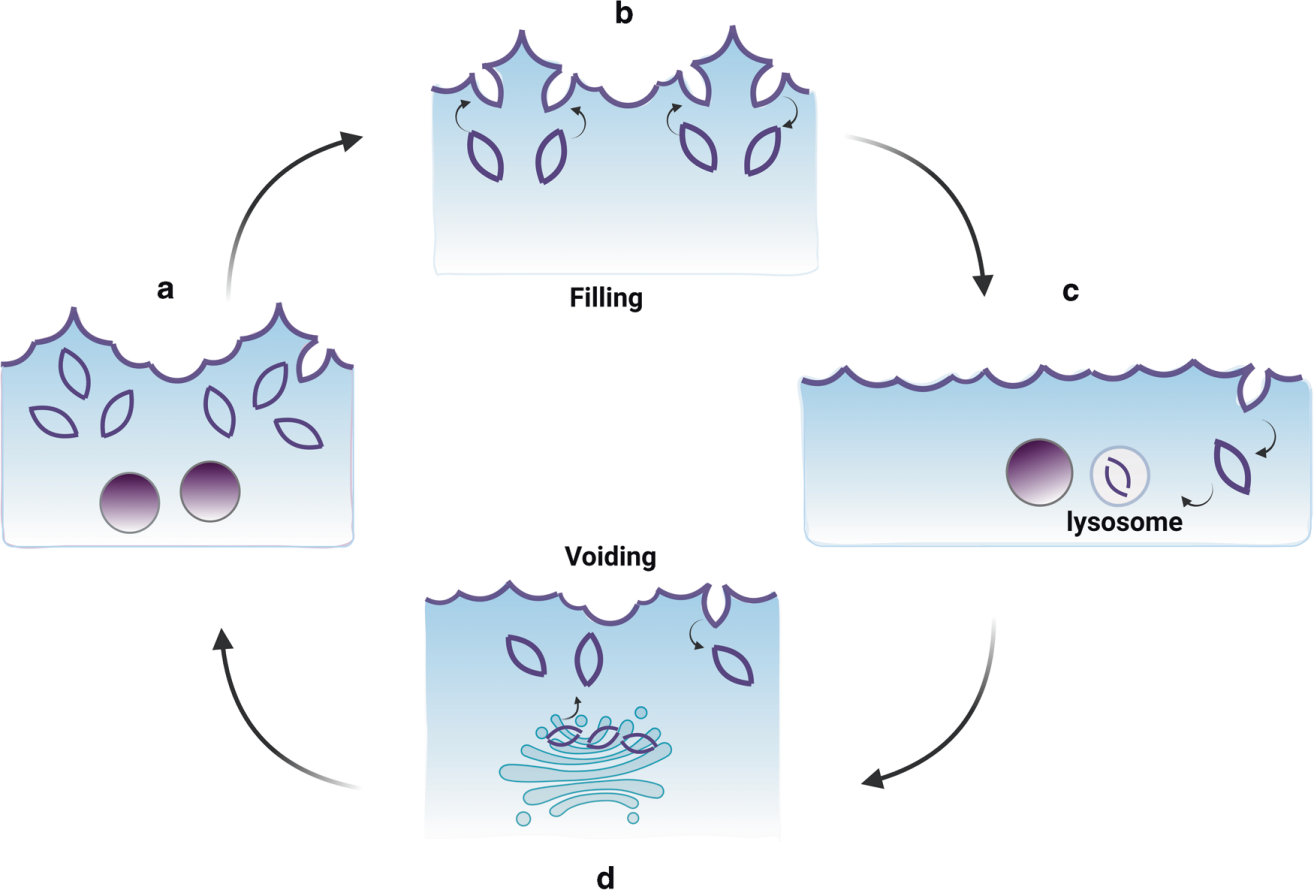
Urothelium expansion and contraction in response to bladder filling and emptying. **a** Umbrella cells in its relaxed/unfilled state, (**b**) the bladder filling stimulates exocytosis of the vesicles coupled with endocytosis, (**c**) the exocytosis of the vesicles leads to increase in umbrella cell apical membrane. The endocytosed vesicles are delivered to lysosomes where the contents are degraded, (**d**) upon voiding, the added apical membrane is internalized, and a new pool of vesicles are formed in the Golgi.

It is thought that maintaining barrier function upon stretching is enabled by a few specializations (Fig. 5). First, the umbrella cell transitions from a parasol shape to a squamous flat form during filling^22^, transitioning from an apical diameter of ~30–50 μm in the relaxed state to ~50–150 μm when stretched. Second, bladder filling triggers a large pool of subapical DFVs to undergo RAB8a, RAB11a, and RAB27b-dependent exocytosis, leading to a dramatic increase of the apical surface area^32–34,36,175–177^. The excess apical membrane is quickly internalized during voiding by an integrin-triggered, dynamin II (DNM2)-dependent, RhoA-dependent, and clathrin-independent, endocytic pathway^32,34,36,178^. The umbrella cell apical junctional ring also plays a critical role in maintaining the urothelial barrier while retaining the structure and function during bladder expansion and contraction. This property is not only limited to umbrella cells, as all epithelial cells are subjected to mechanical stimuli during development and normal physiological functions including lung inflation and fluid flow through nephrons or vasculature^107,150,179–182^.

In addition to these macroscopic changes, other tissue and cell shape changes accompany bladder filling alongside the above-mentioned remodelling of umbrella cells. As the bladder fills, the urothelium thins, and in species with multiple intermediate layers, the urothelium appears to have fewer cell layers^5^. It is surmised, but not yet experimentally determined, that during filling, the intermediate cell layers slide past one another while maintaining their cell-cell contact. Finally, expansion modifies the distribution of proteins associated with the TJ^179,183–185^, and affects junctional strand number and distribution^186–189^.

## Urothelial sensory mechanisms

There is substantial evidence that the urothelium has specialized sensory and signaling properties enabling the urothelial cells to respond to several mechanical or chemical stimuli^190,191^. The urothelium responds to a variety of mechanical stresses during bladder filling and voiding by activating transducer proteins. During the changes in hydrostatic pressure that typically trigger micturition, the urothelial cells release transmitters such as ATP^192^. The urothelium also responds to changes in urine osmolarity. Alterations in urine composition can be viewed as a form of stress, with urine contents varying in both in terms of their delivery rate and their composition^193^.

The bladder urothelium expresses several different receptors and ion channels linked to mechanoceptive or nociceptive sensations^190,191,193–196^. These include purinergic (P2X1-7 and P2Y1, 2, 4)^193,197^, adrenergic (α and β)^193,198^, cholinergic (muscarinic; M1-5 and nicotinic α2-α10, β2 and β4)^193,199,200^, protease-activated receptors^201^, acid sensing ion channels (ASIC)^202^, corticotrophin-releasing factor (CRF1, CRF2)^203^, neurotrophin receptors^204–206^, various transient receptor potential (TRP) channels (TRPV1, TRPV2, TRPV4, TRPM7, TRPM8, TRPA1)^193,194,207–211^, and chemokine receptors such as CXCR4 and CX3CR1^212^. The expression of these receptors and ion channels allows the urothelium to respond to diverse stimuli from a variety of sources. Sensory inputs include stretching and distension during bladder filling^193,194,207–209^, soluble factors found in urine such as nerve growth factor (NGF)^194^, acetylcholine^193,213^, ATP or norepinephrine released from nerves and inflammatory cells, chemokines (CXCL1, CXCL12, CX3CL1, CCL2), which are released from inflammatory cells^212,214,215^, and changes in pH due to inflammation^193,216^. These diverse stimuli can lead to several outputs with complex results including the alteration in the flow of ions and other substances across the urothelium, changes in membrane turnover, and modification of the activity of underlying smooth muscle and neighboring sensory neurons^217^.

Several signalling molecules are secreted by the urothelium, including neurotrophins, neuropeptides, ATP, acetylcholine, prostaglandins, nitric oxide, and cytokines^193,194,209,218^. These molecules can communicate with other cells such as bladder neurons, smooth muscle cells, interstitial cells, and inflammatory cells^193,197^. ATP has been demonstrated to act as a main messenger released from urothelial cells during purinergic mechanosensory transduction, which activates P2X3 receptors indicating bladder fullness, and pain^218,219^.

The bladder also expresses multiple TRP channels from different subfamilies. The TRP comprise a superfamily of nonspecific cationic ion channels that in the urinary bladder are highly expressed in, but not restricted to, primary afferent neurons; they are also expressed in the urothelium and some interstitial cells. Twenty eight TRP channels have been discovered so far in mammals consisting of seven subfamilies: TRPC (canonical), TRPM (melastatin), TRPV (vanilloid), TRPA (ankyrin), TRPP (polycystin), TRPML (mucolipin)^220^, and TRPN (no mechano-potential)^221^. TRP have specific tissue distributions, are activated by many exogenous and endogenous mediators^207,208,222^, and may have functional roles in micturition^223,224^. A number of these channels are also associated with bladder disorders including overactive bladder (OAB) and interstitial cystitis/bladder pain syndrome (IC/BPS)^225,226^.

TRPV1, the first subfamily to be identified, currently includes six members (TRPV1-6). The expression and function of TRPV1 in the urothelium^223,226^, TRPV2 in umbrella cells^227,228^, and TRPV4 in basal and intermediate cells^224,229–233^ are well-documented. The expression of TRPA1^234^ and TRPM8^235^ subfamilies has also been detected in the urothelium.

Mechanosensitive ion channels convert mechanical signals into electrochemical signals and are widely expressed in the urinary system. They are key mechanotransducers in response to stimuli such as shear stress, bladder distension, and emptying the bladder. Piezo1 and Piezo2 are the two family members of Piezo channels expressed in the urinary system^236^. It has been shown that Piezo2 in the lower urinary tract has a dual role, acting as a sensor in both the bladder urothelium and innervating sensory neurons. It has been reported that humans and mice lacking functional Piezo2 have impaired bladder control while humans additionally exhibit deficient bladder-filling sensation^237^. Furthermore, recent evidence also suggests that sensory dysfunction associated with UTI, such as urinary infrequency and pelvic pain, is due to sensitized bladder-innervating sensory afferents caused by the inflammatory events^238^.

## Diseases of the bladder

The bladder mucosa is constantly exposed to microorganisms because of its relative proximity to the gastrointestinal (GI) tract. In addition, in women, the urethral orifice is close to the vaginal mucosa with its own microbiota^239–241^. Urinary tract infections (UTIs) are the most common and frequent infections worldwide, infecting over 150 million people annually^242^ with high treatment costs^242–244^. UTIs can affect the upper (pyelonephritis) or lower (cystitis) urinary tract, with the latter being extremely common, affecting over half of women and 5% of men in their lifetimes^242^. The most frequent bacterial cause of uncomplicated community-acquired UTI is uropathogenic *E. coli* (UPEC), representing over 80% of infections^245^. These bacteria colonize the lower GI tract and can migrate across perineum to the urethra, gaining access to the urinary tract where they can cause disease. Other pathogens associated with uncomplicated UTI include *Staphylococcus saprophyticus, Klebsiella* species, *Proteus mirabilis* and *Enterococcus faecalis*^246^, among many others, including some fungi.

The umbrella cells on the luminal side of the urothelium constitute the first barrier against invading uropathogens, forming a tight monolayer of highly differentiated and polarized cells. The impermeability of umbrella cells plus their protective glycan layer discourages the adherence of bacteria; additionally, the frequent unidirectional flow of urine helps to remove any adherent bacteria, making the urothelium one of the most challenging mucosal surfaces to colonize^247^. There are other factors that can limit urothelial attachment such as changes in urine osmolarity, pH, soluble IgA, uromodulin (Tamm-Horsfall urinary glycoprotein), iron chelating siderophores and antimicrobial peptides (AMPs)^248^.

The urothelium expresses multiple toll-like receptors (TLRs) which recognize pathogen-associated molecular patterns (PAMPs)^249–252^, and damage-associated molecular patterns (DAMPs)^250,252^ generated upon cell or tissue damage^247^. TLR activation triggers the production of inflammatory mediators such as cytokines and chemokines that help to clear infections. The common TLRs identified in the urinary tract include TLR2, TLR3, TLR4, TLR5, TLR9, and TLR11 (the latter in mice only)^253^. Studies have reported that urothelium from normal human bladders express TLR5 (weakly), TLR2, TLR3, and TLR7 (moderately), and TLR4 and TLR9 (strongly)^247,254^.

UPEC infection is initiated by the attachment of *E. coli* to the urothelium via its lectin-type 1-fimbriae (FimH) adhesin, found at the tip of the Type I pilus, to urothelial surface receptor UPK1A, which is rich in mannose residues^255–257^. The reaction is FimH-specific and does not take place with any *E. coli* expressing other types of adhesins or lacking fimbria^258,259^. Upon invasion of the bladder urothelium, uropathogenic bacteria replicate, form intracellular bacterial communities (IBC), and invade neighboring cells^260^. Mouse models show that once within the bladder urothelium, bacteria can survive for long periods leading to recurrent UTIs that are challenging to treat^243,256,257^, although the situation in humans is less well understood.

Infection with UPEC initiates a host response that triggers umbrella cell death and exfoliation to promote bacterial removal^261,262^. But as this manoeuvre exposes the underlying cells to both toxic urine and existing uropathogens in the environment^45^, the underlying cells rapidly proliferate to replace the shed cells within hours^17,40^. UPEC infection also activates TLR4, which is expressed at the apical surface of umbrella cells^263–266^, and its downstream effector myeloid differentiation factor 88 (MyD88) to facilitate bacterial clearance. In Tlr4-deficient mice challenged with UPEC, the infection persists in the bladder and the host exhibits an impaired IL-8 response and ineffective neutrophil mobilization^267^. Pediatric patients with decreased granulocyte TLR4 expression are also more likely to have asymptomatic bacteriuria than those with normal TLR4 expression^268^.

The activation of pattern recognition receptors (PRR) TLR4 and Nod-like receptor/Caspase 1 lead to the secretion of IL-6 and IL-1β which are detectable in urine^269,270^. The expression of cytokines along with other inflammatory mediators secreted by urothelial cells result in the influx of immune cells to the site of infection. Bladder urothelial cells secret several AMPs that complement the cytokine responses such as cathelicidin LL-37^271^ and β-defensin; although this latter AMP is found in urine, it mainly originates from kidney epithelial cells^272^. Both LL-37 and β-defensin also contribute to cytokine production and neutrophil recruitment in the bladder^273^. Ribonuclease 7 is another AMP that has broad-spectrum microbial activity against many uropathogens^274^. The soluble pattern recognition molecule pentraxin-related protein 3 (PTX3) is thought to lead to complement-mediated killing by binding to bacterial surfaces, and increasing bacteria uptake by phagocytes^275^. In humans, increased UTI incidence is correlated with mutations in the PTX3 locus^276^.

The urothelium not only forms a highly effective barrier to urine and pathogens, and functions as a source of soluble AMPs, but it also performs a critical role in regulating bladder volume in the course of urine filling and emptying. Unfortunately, this process can be hijacked by invading bacteria. The urothelium contains a large number of RAB27b^+^ DFVs^175^. As mentioned previously, when the bladder fills, extra membrane is provided by DFVs, which spontaneously exocytose into the plasma membrane in a cyclic AMP (cAMP)-dependent manner. After the void, the intracellular DFVs form once again facilitate urothelial contraction by internalizing the RAB27^+^ membranes^175,277^. Following UPEC binding to the apical surface of the urothelium, TLR4 signalling leads to increased intracellular levels of cAMP, which consequently triggers spontaneous expulsion of RAB27b^+^ vesicles at the adherence site. Subsequently, the RAB27b^+^ vesicles retract from the cell surface and draw the invading bacteria back into the cells with them, encased in RAB27b^+^ vesicles^275,277^. The urothelium has a defense system capable of sensing the invading bacteria and initiates mechanisms to expel the intracellular bacteria. This activity is triggered by TLR4 localized in the vesicles encapsulating the bacteria and is initiated within a few minutes of bacterial entry^275^.

Remarkably, not all intracellular bacteria are exocytosed from RAB27b^+^ vesicles and a considerable number of UPEC escape intracellular vesicles and enter the cytosol. A study showed that UPEC initiates escape by upregulating phospholipase PIdA upon sensing host immune responses. UPEC infection upregulates *PIT1*, a host phosphate transporter located on the vesicle membrane, via NF-κB, resulting in phosphate reduction which in return activates the expression of *pldA* to disrupt the vesicle membrane^278^. A second exocytic pathway is activated by the cell autophagy system which recognizes and captures the bacteria in autophagosomes and transports them to the lysosome. It has been shown that mice hypomorphic for ATG16L1 have reduced UPEC persistence. Furthermore, network mapping of autophagy pathways has identified RAB33b, a Golgi-resident small GTPase, which interacts directly with ATG16L1 modulating autophagosome formation. Small RAB GTPases (RAB27b and RAB11a) are highly expressed in umbrella cells and are key for vesicle trafficking, UPK recycling and exosome-mediated intracellular UPEC expulsion^279^. In addition, UPEC co-opts ferritinophagy (a selective form of autophagy) and shuttles into the autophagosomal and lysosomal compartments with ferritin-bound iron, facilitating UPEC survival and persistence within the urothelium^280^. Autophagy usually leads to bacterial degradation, but UPEC can block acidification and survive within lysosomes^281^. Studies using cultured human bladder cells have shown that the malfunctioning lysosomes containing UPEC are rapidly sensed by TRP mucolipin 3 (TRPML3), a cation channel expressed on the lysosomes. TRPML3 is activated when the pH within the lysosome increases, triggering the spontaneous exocytosis of these lysosomes^281^. Contrary to the first wave of bacterial expulsion, bacteria expelled from lysosomes in this manner are encased within host membranes, preventing re-attachment of UPEC to the urothelium and ensuring bacterial removal in urine^281^.

Another defense mechanism employed by the urothelium to reduce bacterial load is undergoing cell death and cell exfoliation into the urine, thus eliminating the cells that are associated with adherent and intracellular bacteria^261,262^. This allows the removal of large numbers of bacteria but consequently exposes the underlying cells to both toxic urine and existing uropathogens in the environment^45^. It has also been demonstrated that the NF-E2-related factor 2 (NRF2) pathway is activated in response to UPEC-triggered reactive oxygen species (ROS) production. The NRF2 activation in urothelial cells causes the reduction of ROS production, inflammation, and cell death resulting in UPEC expulsion and a reduction in bacterial load^282^.

### Lower urinary tract symptoms

Lower urinary tract symptoms (LUTS), such as frequency, urgency, and dysuria, are particularly prevalent among adults. Patients with isolated or repeated episodes of LUTS with positive urine cultures are often treated with short courses of antibiotics. However, no aetiology is found for many LUTS patients with negative results using standard urine culture techniques, and no abnormal functional or anatomical urinary tract, although it should be noted that traditional tests have been shown to be insensitive and miss genuine infections^283^. Patients with urgency as their main complaint and no signs of infection are often diagnosed with overactive bladder (OAB), while patients with pain, pressure, or discomfort are diagnosed with interstitial cystitis/bladder pain syndrome (IC/BPS)^284^.

IC/BPS is estimated to affect 3–8 million women and 1–4 million men^285^. The difficulties in diagnosis originate not only from insensitive UTI tests, but also from many theories regarding pathophysiology and aetiology such as diminished GAG layer, altered permeability of the urothelium, uroinflammation, and neural upregulation^286^. Studies have reported a decreased amount of GAG in the urine of patients with IC/BPS which has also been confirmed in animal models^287,288^. Another study examined tissue from bladder biopsies of patients with IC/PBS and observed considerable abnormalities in the level of CK18 (80% showing abnormalities), CK20 (87.5%) and uroplakins (56%)^289^. Studies of rodent models of IC/BPS also reported lower expression of urothelial surface proteins, their incorrect arrangements^290^, and damage of TJs seen with electron microscopy^291^. In patients with non-ulcerative IC/BPS, a more common IC/BPS accounting for almost 90% of all cases^285^, a lower amount of UPK1A, UPK1B, and UPK2 mRNA expression were detected in bladder tissue apart from UPK3^292^. UPK3-delta4, a splicing variant of UPK3, was significantly upregulated in IC samples, which has been suggested as a promising marker of IC/BPS. Further research is required to understand the etiology of OAB and IC/PBS, with the caveat that the development of more sensitive detection methods for UTI might well reveal an infective element for some unknown proportion of these cases.

## Urothelial in vitro models to study host-pathogen interactions

Animal models provide an invaluable insight into disease pathogenesis. Although murine models of UTI remain indispensable, there are concerns that they do not always fully recapitulate the human tissue environment to correctly predict disease physiology or prospective treatments^293,294^. In such cases, the availability of suitable ex vivo or in vitro human urothelial culture models are beneficial to gather specific insights which could provide critical information.

Traditionally, two-dimensional (2D) monolayer cultures on flat or rigid surfaces have been used for cell-based studies and have proven to be a valuable method. However, their limitations have been increasingly recognized. As most cells in vivo are surrounded by extracellular matrix (ECM) and other cell types in a three-dimensional (3D) manner, 2D cultures do not sufficiently represent the normal 3D environment. As a result, 2D culture experiments sometimes result in misleading or unpredictive data for in vivo responses^295–297^. In contrast, recent studies have suggested that 3D cell culture systems, despite their obvious limitations such as lack of a blood supply or systemic responses, represent a more accurate, tissue-like microenvironment, and may be more reflective of in vivo cellular responses. Research has revealed that cells in a 3D culture environment differ morphologically and physiologically from cells in 2D cultures^298–300^. It is thought that the additional dimensionality of 3D cultures is the crucial attribute that leads to at least some key differences in cellular responses by conferring more reminiscence spatial and physical aspects of the culture^301–303^.

Human urothelial cultures are widely represented in vitro as immortalized or cancer-derived cell lines. However, normal immortalized cells are compromised in their ability to undergo cell differentiation and barrier formation^304^, while the cancerous nature of established cell lines is also a drawback. Non-transformed NHU cells grown as monolayer cultures have been used extensively in many studies^305,306^. However, their rapid loss of quiescence and differentiation characteristics soon leads to a highly proliferative and non-specialized phenotype which governs their response^307–309^.

Arguably, the most biologically and structurally relevant in vitro model of the urothelium is provided by ex vivo cultures of the urothelium^310,311^, where the intact tissue retains most of its in situ tissue architecture. An alternative approach is explant culture, in which primary cultures are established from tissue fragments^312^; however, the use of human organ or explant culture is largely hampered by the limited tissue supply^313^. A “biomimetic” urothelial cell culture system propagated from normal human urothelial (NHU) cells have been described previously^307,314^, exhibiting barrier formation and a multilayer epithelium^315^. The authors demonstrated morphological similarities between the developed “biomimetic” urothelial model and the naïve tissue.

The implications associated with the use of non-transformed normal urothelial cells such as ethics, finite lifespan, and donor variability can be hindrance for many studies. An alternative strategy to extend the lifespan of urothelial cells is immortalization by targeting the telomerase via overexpression of the catalytic subunit of human telomerase reverse transcriptase (hTERT)^316,317^. This would maintain the in situ representation of primary cells combined with the in vitro immortality of cancer cell lines^317,318^. However, studies showed that hTERT-imortalization of human urothelial cells negatively impacts the differentiation or barrier forming capacity of the cells and thereby reducing their biological relevance^304,317^. On the other hand, our group has published a stratified, urine-tolerant biomimetic model derived from commercially available bladder progenitor cells which, despite being spontaneously transformed, still retain the ability to terminally differentiate^319^. The most recent version, 3D urine-tolerant human urothelial (3D-UHU), has been improved to offer a homogenously differentiated umbrella cell layer, excellent barrier function and the ability to secrete key cytokines and chemokines in response to infection^320^.

As an alternative approach, several studies have demonstrated that large number of viable normal urothelial cells can be generated from patient/donor urine samples^321,322^, or bladder washing^313,323,324^. Nevertheless, caution is warranted as these cultures represent an epithelial cell population derived from various regions of the urinary tract and mainly from the kidneys^325,326^. Besides, it is unlikely that sufficient numbers of normal urothelial cells are voided in the urine, considering the longevity and low turnover of the urothelium. Nevertheless, one study did identify urine-derived stem cells that were a subpopulation of urine-derived stem cells with multipotent differentiation capacity^327,328^.

One way to harness the impressive self-renewal capacity of urothelial cells in vivo is to find the source of progenitor or stem cells that self-renew, regenerate, and differentiate in situ; however, as alluded to previously, the origin of urothelial stem cells is a subject of debate^16,17,40,47,49,316,329,330^. 3D organotypic cell cultures derived from primary tissues (either tissue subunits or single cells), adult stem cells, fetal/postnatal stem cells, pluripotent embryonic stem cells (ESCs) and induced pluripotent stem cells (iPSCs) offer possibilities of studying the urothelial cells in a more in vivo like condition. Adult stem cells are an attractive source for bioengineering a urothelium as they are relatively easy to obtain and culture and are autologous^327,331,332^. Fetal or postnatal stem cells have also been differentiated into urothelium^333–335^.

However, both adult and fetal stem cells are limited by their poorly understood differentiation processes. Furthermore, adult stem cells have limited proliferation potential in vitro, and fetal cells have possible immunological consequences if being considered for regenerative medicine. Therefore, the pluripotent nature of ESCs and iPSCs make them attractive candidates. In a study using human PSC, cells were differentiated into bladder urothelial cells which expressed several marker genes such as uroplakin and cytokeratin; also, they formed a terminally differentiated monolayer. However the resulting cell layer stratification was not comparable to that of native urothelium^336^. In another study, 3D bladder ‘assembloids’, organoids derived from normal urothelial stem cells or patients with bladder tumors, were reconstituted with stromal components. They manifested an organized structure with an epithelium surrounding stroma and an outer muscle layer. The assembloids exhibited mature adult bladder characteristics in cell composition and gene expression at the single-cell level; furthermore, they demonstrated regenerative responses to injury mimicking in vivo tissue dynamics^337^. Apart from studies published by our group^319,320,338^, most described models either were not exposed to urine or only for short periods^339–342^. This is potentially important as urine has an effect not only on human cell physiology, but also, in the case of UTI research, on bacterial behavior^343,344^.

In vitro modelling for bladder cancer-studies is a vibrant and expanding area but is beyond the scope of this review. These traditionally employ bladder carcinoma cells grade 1–4^345^, but exciting inroads have also been made into personalized, patient-derived bladder cancer organoids^346^.

## Conclusions and future perspectives

The urothelium is a unique epithelial surface comprised of multiple cell layers. It can change size and shape to accommodate fluctuating volumes of urine and simultaneously provide a barrier to prevent absorption of toxic substances and to defend against microbial entry. Bladder dysfunction and urinary tract chronic diseases significantly impact quality of life for millions of people worldwide. Although much has been learned over the past decades, a number of unknowns remain to be elucidated, including how the urothelium differs with age; sex differences in basic biology and disease outcomes; integration of urothelial function with neuronal signaling; and urothelial-immune interactions. While rodent models have significantly improved our understanding of urinary tract disease pathogenesis, the considerable structural and biological differences between species calls for the development of alternative models. In the past few years, an immense effort has been dedicated to the development of a variety of human-based 3D culture systems as well as adoption of these models in drug discovery, cancer cell biology, stem cell biology, and in efforts to engineer functional tissues for implantation, among other cell-based research. Such 3D culture models provide suitable in vitro systems to study cellular responses in a setting that mimics the in vivo microenvironment^295,347–349^. In addition, recent developments of organoids co-cultured with immune or rare cell types have significantly improved our understanding of the dynamic interactions between complex tissues and different cell types within a controlled environment.

Casting ahead to the future, we look forward to advances in human cell-based model technologies that overcome the biggest limitations of the current platforms, namely lack of a systemic environment to provide the necessary crosstalk. Once perfected, Microfluidic Organ Chip (Body-on-a-Chip) systems could allow communication between existing bladder microphysiological platforms alongside mechanical stimuli, vascular components, ECM, circulating immune cells and even resident microbial communities^350^. There has already been one report of an unstratified human carcinoma cell line-derived urothelial model cultured adjacent to endothelial cells, which also contains flow and mechanical stretch components^351^; combining this idea with a fully stratified non-cancer-derived urothelial model and further systemic elements would be the next logical step. Such fully integrated systems should be essential as complementary tools alongside both animal and clinical studies in patients to fully understand normal bladder physiology as well as how it can go wrong in infection, injury or disease.
